# Fortifying the Treatment of Prostate Cancer with Physical Activity

**DOI:** 10.1155/2016/9462975

**Published:** 2016-02-10

**Authors:** Colin E. Champ, Lanie Francis, Rainer J. Klement, Roger Dickerman, Ryan P. Smith

**Affiliations:** ^1^Department of Radiation Oncology, University of Pittsburgh Medical Center, Pittsburgh, PA 15215, USA; ^2^Department of Integrative Oncology, University of Pittsburgh Medical Center, Pittsburgh, PA 15232, USA; ^3^Department of Medical Oncology, University of Pittsburgh Medical Center, Pittsburgh, PA 15232, USA; ^4^Department of Radiation Oncology, Leopoldina Hospital, 97422 Schweinfurt, Germany; ^5^Relentless Fitness, Philadelphia, PA 19106, USA

## Abstract

Over the past decade, significant data have shown that obese men experience a survival detriment after treatment for prostate cancer. While methods to combat obesity are of utmost importance for the prostate cancer patient, newer data reveal the overall metabolic improvements that accompany increased activity levels and intense exercise beyond weight loss. Along these lines, a plethora of data have shown improvement in prostate cancer-specific outcomes after treatment accompanied with these activity levels. This review discusses the metabolic mechanisms in which increased activity levels and exercise can help improve both outcomes for men treated for prostate cancer while lowering the side effects of treatment.

## 1. Introduction: Prostate Cancer, Obesity, and Metabolic Health

In 1985, the Radiation Therapy Oncology Group (RTOG) set out to examine the benefit of hormonal therapy in the treatment of prostate cancer. RTOG 85-31 randomized 945 men with locally advanced prostate cancer to radiation therapy (RT) and immediate (concurrent) versus delayed androgen deprivation therapy (ADT) [1]. The study revealed a benefit with the addition of immediate ADT, which is now the standard of care for men undergoing definitive RT for high-risk prostate cancer. Several other studies have revealed similar survival benefits with the addition of ADT to RT [2, 3]. Interestingly, subset analysis of long-term results from yet another positive ADT study revealed that those patients without a history of comorbid illness, such as myocardial infarction or diabetes, may not derive similar benefits [4].

Both RT and ADT work by interfering with tumor cell replication. RT primarily inflicts tumor cell injury through both direct and indirect DNA damage via the generation of free radicals. The mechanisms with which ADT treats prostate cancer remain more elusive, and it is thought to work primarily by reducing the transcription of genes involved in cell-cycle regulation and proliferation [5]. Circulating androgens like testosterone and dihydrotestosterone (DHT) bind to the androgen receptor on prostate gland and prostate cancer cells, leading to gene transcription. ADT is achieved via medical or surgical castration to reduce levels of circulating androgens. In combination, it is felt that ADT can sensitize cells to enhance damage from RT.

While RTOG 85-31 revealed a benefit to the addition of immediate ADT, further analysis of the dataset revealed that those patients with a body mass index (BMI) of 30 or more had a significant detriment in prostate cancer-specific survival [6]. This was one of the first major randomized trials to illustrate the importance of a healthy metabolic state for men with prostate cancer during and after treatment. Other epidemiologic data have confirmed this relationship [7] and revealed an increase in prostate cancer metastases in obese men [8]. In a meta-analysis published in 2011, it was estimated that for every 5 kg/m^2^ increment in BMI there was a 21% higher risk of biochemical recurrence and a 20% higher risk of prostate cancer-specific mortality [9].

Although BMI cannot provide an exact quantification of muscle, bone, and adipose tissue, it has been shown to be useful as a crude measure of excess adiposity. As such, BMI is correlated with several other physiologic factors characteristic of metabolic dysregulation and metabolic syndrome [10]. Metabolic syndrome, also known as insulin insensitivity syndrome, is defined as central obesity in addition to two of the following risk factors: elevated glucose, insulin resistance, elevated triglycerides, reduced high-density lipoproteins (HDL), and hypertension [11]. This metabolic state has been shown to potentially provide cancer cells with an enhanced ability to withstand damage from RT [12], while obesity leads to a state of alteration of testosterone, estrogen, insulin, insulin-like growth factor-1 (IGF-1), and leptin, all hormones linked to prostate cancer, which could potentially interfere with hormonal therapy [13]. Along these lines, several reasons for the correlation of poorer outcomes for men with prostate cancer who also have a surplus of adipose tissue exist and will be discussed below. 

*Inflammation and Adipose Tissue as an Endocrine Organ*. Obese patients experienced poorer outcomes in RTOG 85-31. Similar findings have revealed worse outcomes in obese men treated with prostatectomy for their prostate cancer [14, 15]. In these studies, obese men were found to have higher grade tumors, higher biochemical failure rates, and an increased risk of positive margins after surgery. Additional data reveal that adipose tissue acts as an endocrine organ to secrete inflammatory hormones called adipokines and is associated with insulin resistance [16]. Insulin resistance leads to elevating levels of circulating insulin, serum glucose, and inflammation, all factors which can fuel cancer progression, along with weight gain and poorer responses to cancer treatment [17].

**Figure 1:**
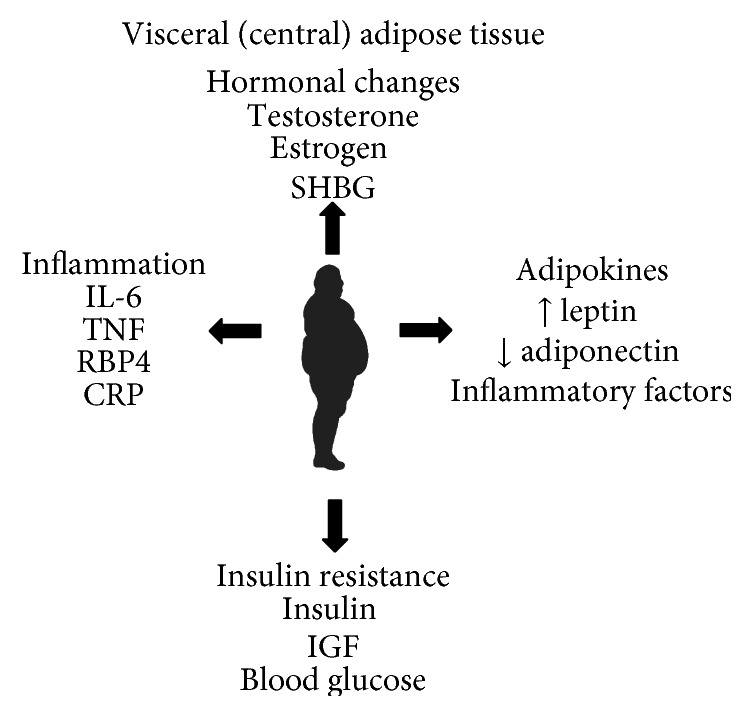
Central obesity leads to the secretion of multiple inflammatory mediators that can worsen prostate cancer-specific outcomes.

Newer studies have implicated central obesity and waist circumference, as opposed to BMI, as the culprit that leads to obesity-related health risk due to the physiologic mechanisms by which adipose tissue acts as an endocrine organ, leading to global metabolic dysfunction [18]. As men tend to accumulate adipose tissue centrally, this is a concern in the prostate cancer patient (refer to Figure 1).

Several inflammatory effects result from excess adipose tissue via the secretion of adipokines. These include dysregulation of cellular growth, angiogenesis stimulation, and extracellular matrix remodeling favoring tumor progression and recurrence [19]. Fat cells secrete the inflammatory mediators tumor necrosis factor-alpha (TNF-*α*) and interleukin 6 (IL-6), which promote cancer induction [20]. Both have been associated with shorter survival, worse disease, and metastases in men with prostate cancer [21]. The third common inflammatory factor released by adipose tissue is C-reactive protein (CRP) [22]. CRP is associated with both obesity and central adiposity and predicts for poor outcomes in men with metastatic prostate cancer, independent of their serum PSA [23]. Similar results have been seen with breast cancer survivors, as discussed previously [17].

Finally, an increased inflammatory cytokine profile has been linked to cancer related fatigue (CRF) [24], a state characterized by overall weakness and increased need for sleep and rest. Greenberg et al. showed that symptoms of CRF increased during the course of RT in prostate cancer patients independent of depressive symptoms but connected to changes in serum IL-1 levels [25]. This provides a plausible mechanism for adiposity augmenting treatment-induced fatigue and increasing the risk for prolonged and more severe posttreatment CRF [26].

*Hormonal Production of Adipose Tissue and Insulin Dysregulation*. Excess adipose tissue works though many indirect mechanisms to cause insulin insensitivity and chronically elevated levels of serum glucose, which can lead to cancer progression and resistance to cancer treatments. One direct mechanism is through the release of a hormone known as resistin (resistance to insulin), which impairs glucose tolerance and the action of insulin to lower blood glucose levels [27]. Adipose tissue accumulation also leads to elevated levels of plasma free fatty acids, which inhibit the normal physiologic uptake of peripheral glucose via insulin stimulation. This potentially occurs via the inhibition of glucose transport, via a decrease in muscle glycogen synthase activity, or via the stimulation of insulin secretion, ultimately leading to insulin insensitivity and hepatic glucose overproduction [28].

Dietary-induced hyperinsulinemia via excessive consumption of carbohydrate food sources has been shown to increase levels of IGF-1 and activate the insulin pathway and AKT, increasing prostate cancer growth in mouse studies [29]. Other studies reveal that glucose in itself can bind and activate the insulin receptor and pathway [30], fueling cancer growth and proliferation, while aiding in the repair of tumor damage from RT [12]. Accordingly, the uptake of the glucose analog tracer ^18^F-fluorodeoxyglucose in preoperative positron emission tomography scans has been shown to predict for prostate cancer stage and 5-year progression free survival after radical prostatectomy [31].

Activation of the insulin pathway can lead to cancer progression and resistance to current treatment modalities, including RT [32, 33]. IGF-I upregulates the insulin pathway, stimulating the growth and progression of prostate cancer cells [34]. DHT appears to work synergistically with IGF-1 to enhance prostate cancer progression. Conversely, insulin-like growth factor binding proteins (IGFBPs) can bind and inactivate IGF-1 to offset its potentially negative effects on cancer outcomes [35]. IGFBP-3 has specifically been shown to induce apoptosis in prostate cancer cells [36]. Accordingly, Rundqvist et al. have shown that serum taken from male subjects after intense exercise inhibited growth of prostate cancer cell lines in SCID mice through an increase in IGFBP [37]. Obese individuals have lower levels of IGFBP-1 and IGFBP-2, with saturation of IGFBP-3, and subsequently higher levels of IGF-1 [38].

Sex hormone-binding globulin (SHBG) works similar to ADT by endogenously binding to circulating DHT and testosterone to reduce their bioavailability to bind to prostate cells. However, serum insulin inhibits SHBG production within the liver. In this regard, insulin and BMI are inversely related to SHBG [39].

Minimizing excess adipose tissue and the reduction of blood glucose and insulin levels may be a potent method of reducing prostate cancer risk and improving outcomes.

## 2. Activity Levels and Exercise: Metabolic Modification to Improve Prostate Cancer Outcomes

A prudent method to increase patient outcomes would thus incorporate techniques to mitigate levels of circulating glucose and insulin, reduce excess adipose tissue, limit inflammation, and optimally balance hormonal levels. Exercise is generally felt to improve global metabolic status. As discussed below, a plethora of data have linked activity levels with positive prostate cancer-specific outcomes. The exact activities that lead to the largest benefit remain unknown, and data generally and nearly unanimously reveal that increased overall activity levels provide overall and prostate-specific health benefits.

In a study following over 2,000 men with prostate cancer, it was found that men who were more active lived significantly longer [40]. Furthermore, men who walked 90 or more minutes per week at a brisk pace experienced half the risk of dying versus those who did not walk or did so at a slow pace. Three or more hours per week of vigorous activity was associated with a 61% decreased risk of dying from prostate cancer. Finally, men who exercised vigorously before and after their diagnosis had the lowest risk of dying from prostate cancer.

Other data have paralleled the importance of more vigorous activity. In a dataset of 1,455 men diagnosed with clinically localized prostate cancer, those who walked at a pace of over 3 miles per hour had a 57% lower rate of progression than those who walked at a slower pace for under three hours per week. This benefit was also independent of duration [41]. The authors went as far to suggest that “Brisk walking after diagnosis may inhibit or delay prostate cancer progression among men diagnosed with clinically localized prostate cancer.”

Recent studies have begun to parse the benefits of specific activities. In a cohort of 4,623 men diagnosed with localized prostate cancer, a 37% reduction in overall mortality rates was seen in those men who engaged in five or more metabolic equivalent tasks (MET) [42]. Men who walked or bicycled for 20 or more minutes per day experienced a 30% reduction in overall mortality and those who exercised for an hour or more per week had a 26% reduction. Interestingly, men who performed an hour or more of household work per day also experienced a 29% reduction in overall mortality. While briskness holds importance, there appears to be a variety of activities that can provide significant benefit. These activities have as significant an effect on prostate cancer-specific mortality (PCSM) as well; men who walk or ride a bike for 20 or more minutes per day experience a 39% reduction in PCSM and men that exercise for an hour or more per week have a 32% reduction in PCSM.

## 3. Physiological Benefits of Physical Activity

While data is mixed, exercise has generally been considered to result in weight loss and, specifically, lower amounts of adipose tissue [43, 44]. Indeed, a reduction in adipose tissue serves to eliminate several heads of the metabolic hydra seen with central obesity. However, the major benefits of exercise may consist of the metabolic alterations that accompany increased activity levels and specifically brisk and intense activities.

A single bout of high-intensity exercise results in the breakdown of glucose and muscle glycogen, significantly lowering serum glucose levels and enhancing insulin sensitivity [45]. Such effects may have little impact on acute weight loss but would result in metabolic alterations favoring a more inhospitable environment for tumor cells, especially during treatment with RT. Furthermore, even a single bout of low-intensity exercise leads to metabolic alterations, including the enhancement of insulin sensitivity and breakdown of fatty acids that persist for the following day [46].

The intensity level of exercise may lead to different benefits. With regard to intense exercise, described as “briskness” in the studies mentioned above, serum glucose uptake and glycogen oxidation are increased [47], thus improving glucose and insulin-based metabolic dysfunction. Lower intensity exercise may lead to more beneficial effects with regard to the reduction of adipose tissue; data reveal maximal peripheral lipolysis and fatty acid release from low-intensity exercise. The body appears to shift to triglyceride lipolysis when intensity is increased, which would further affect one of the hallmarks of metabolic syndrome. Thus, it appears that there are significant benefits from both intense activities like weight lifting and sprinting along with less intense activities like walking, riding a bicycle, or even performing household chores, as described by Bonn et al. [42].

## 4. Muscle Mass and Mitochondrial Biogenesis

**Figure 2:**
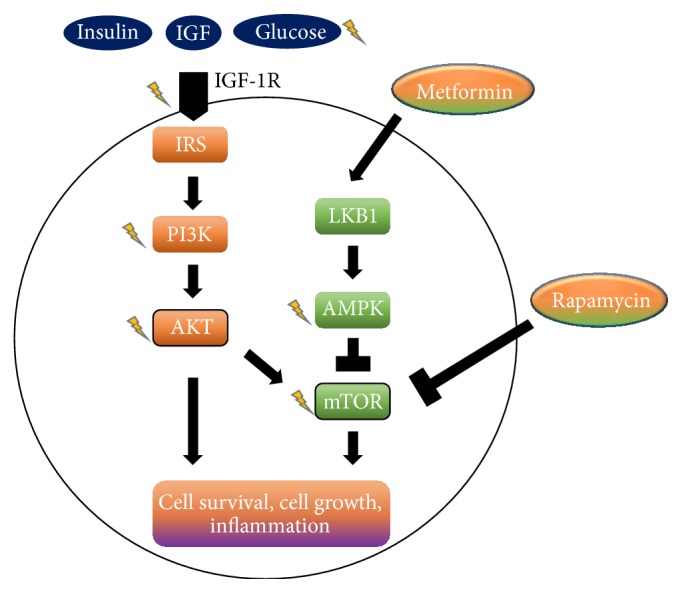
Metabolic manipulation of the AMPK overlaps with activation via exercise. Lightning bolts indicate pathways that affect radiosensitivity. AMPK, AMP-activated protein kinase; IGF-1, insulin growth factor-1; IRS, insulin receptor substrate; LKB1, liver kinase B1; mTOR, mammalian target of rapamycin; PI3K, phosphatidylinositol 3-kinase. Image is used with permission from Champ et al., 2013 [33].

Just as adipose tissue works as endocrine organ, muscle tissue appears to work in a nearly opposite manner to release factors that counter inflammation. When muscle contraction occurs during activity and exercise, adenosine triphosphate (ATP) is consumed for energy derivation. As the intracellular ATP/AMP ratio is reduced, there is cellular activation of the liver kinase B1- (LKB1-) adenosine monophosphate-activated protein kinase (AMPK) pathway. AMPK inhibits the mammalian target of rapamycin (mTOR) protein, which has been implicated in prostate cancer cell progression [48] and is a current target of prostate cancer treatment [49]. Elevation of the insulin pathway, on the other hand, reverses the antitumor effects of inhibition of the mTOR pathway [49] (Figure 2).

Studies assessing human muscle after exercise reveal increased levels of AMPK expression with intense exercise [50], and powerful muscle contraction results in the potent stimulation of AMPK [51]. Interestingly, in those individuals who exercise frequently, AMPK protein levels remain elevated in skeletal muscle afterwards during periods of inactivity [52]. Perhaps most importantly for the prostate cancer patient with diabetes or some degree of insulin insensitivity, muscle contraction-stimulated release of AMPK and the mitigation of serum glucose levels via cellular influx are independent of insulin sensitivity [53]. Furthermore, muscle contractions and activation of AMPK result in translocation of the GLUT-4 receptor in myocytes, leading to glucose influx and the lowering of serum glucose levels [54], which would have a favorable impact on metabolic syndrome, serum insulin levels, inflammation, and even obesity.

In contrast to the ample data in skeletal muscle, data regarding exercise modulation of AMPK levels or phosphorylation in prostate tumors remain elusive. One study using a murine breast cancer model found no differences in AMPK protein expression between tumors from wheel-running and sedentary animals, but contrary to other tumor models these tumors also did not differ in growth rates [55]. More data exist regarding an effect of exercise on AMPK via modulating adiponectin levels which correlate negatively with obesity and increase moderately during various exercise regimes [56]. AMPK activation by adiponectin has been shown to inhibit prostate and colon cancer cell viability [57] but paradoxically also enhanced prostate cancer cell migration and metastatic potential [58]. Adding to this controversy, Rider et al. recently found that high expression of the adiponectin receptor 2 in prostate tumors was associated with increased proliferation and worse survival but was not associated with BMI or PSA levels [59]. The role of exercise-induced AMPK activation in prostate cancer therefore remains somewhat speculative, while the metabolic benefits of global upregulation of AMPK remain clearer.

Faubert et al. have shown that stimulation of AMPK suppresses tumor growth, the uptake of glucose, and aerobic glycolysis of tumor cells, known as the Warburg effect [60]. Their data also revealed that the activation of AMPK serves to downregulate HIF-1*α*, which can potently increase the radiosensitivity of tumor cells [61]. Metformin has similarly been shown to increase AMPK and enhance radiosensitivity of tumor cells [62] and is now being assessed in clinical trials [63]. Other data reveal that the activity of AMPK directly increases RT efficacy and regulates tumor survival after irradiation [64, 65]. Hence, its effect on prostate cancer cells certainly may be similar.

While activation of the AMPK pathway may have direct antitumor effects, the global metabolic changes may indirectly affect cancer treatment and outcomes. AMPK activation results in the oxidation of lipids and an increase in the ratio of NAD+/NADH, enhancing metabolism via the upregulation of the NAD+-dependent deacetylase silent mating type information regulation 2 homologue 1 (SIRT1) [36]. This pathway affects cellular metabolism via epigenetic alterations on gene transcription and protein modification. Further along, this leads to mitochondrial biogenesis [37]. Preclinical data have revealed that mitochondrial biogenesis and upregulation alone may have antitumor properties [66, 67].

**Figure 3:**
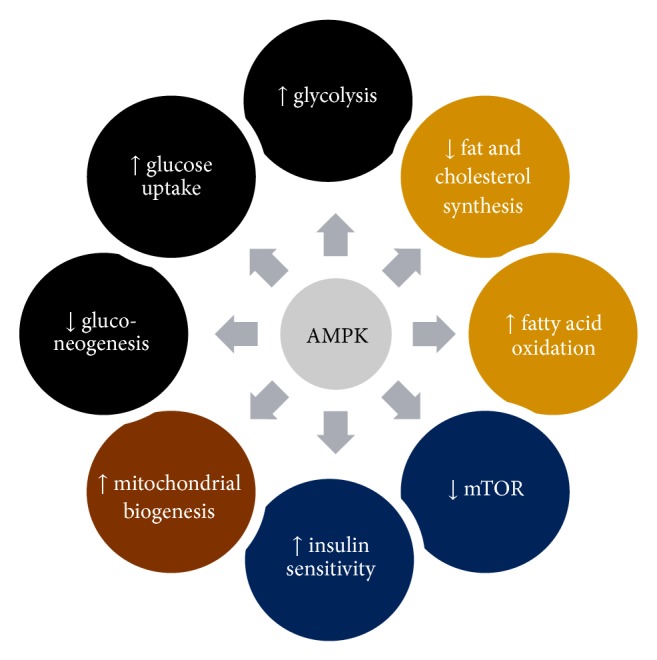
AMPK activation leads to multiple metabolic alterations.

During the generation of ATP, AMPK promotes the breakdown of glucose, glycogen, and fatty acids while inhibiting anabolic processes such as the synthesis of cholesterol, triglycerides, or fatty acids [68] (refer to Figure 3). As tumor metabolism is largely dependent on glycolysis and multiple studies have revealed poorer outcomes with elevated levels of glucose [17], exercise and AMPK activation may be one method to combat the glycolytic phenotype of most cancers.

The data correlating briskness and resistance training with increased benefits may be due to the recruitment of muscle activation during intense activity or heavy lifting. Quite opposite of adipose tissue, the stimulation of muscle releases myokines that appear to lower systemic inflammation [69]. Studies assessing human muscle after exercise reveal increased levels of AMPK expression with intense exercise [50], and powerful muscle contraction results in the potent stimulation of AMPK [51]. Interestingly, in those individuals who exercise frequently, AMPK protein levels remain elevated in skeletal muscle afterwards during periods of inactivity [52]. Perhaps most importantly for the prostate cancer patient with diabetes or some degree of insulin insensitivity, muscle contraction-stimulated release of AMPK and the mitigation of serum glucose levels via cellular influx are independent of insulin sensitivity [53]. Furthermore, muscle contractions and activation of AMPK result in translocation of the GLUT-4 receptor in myocytes, leading to glucose influx and the lowering of serum glucose levels [54], which would have a favorable impact on metabolic syndrome, serum insulin levels, inflammation, and even obesity.

Intense activity may have a more potent effect on lowering increased insulin sensitivity, thus decreasing systemic levels of insulin and serum glucose. General exercise lowers systemic inflammation [70], and the stimulation of one of the best-studied inflammation-modulating myokines is IL-6. While adipose tissue-derived IL-6 and IL-6 produced by macrophages have proinflammatory effects, muscle-derived IL-6 appears to have anti-inflammatory properties [69]. Up to 100-fold transcription of the muscular IL-6 gene occurs after 30 minutes of exercise and has been confirmed with muscle biopsies [71]. This myokine counteracts the proinflammatory action of TNF-alpha [72], which is associated with significantly worse outcomes in men treated for prostate cancer [21]. Furthermore, it has been postulated that muscle-derived IL-6 helps against CRF by decreasing levels of IL-1 and TNF-alpha and elevating levels of cortisol, which in itself has anti-inflammatory effects [73]. Thereby a chronic exercise routine of longer low-to-moderate exercise intermixed with short intense bouts that stimulate muscular contraction may be preferred over infrequent prolonged and/or strenuous sessions; the former may improve the tumoricidal action of macrophages while promoting an overall anti-inflammatory state, while the latter may augment inflammatory and fatigue signaling to the central nervous system [74]. In particular, data indicate that patients at risk for CRF during treatment should refrain from protracted high-intensity competitions such as ultraendurance races as these have been shown to result in a prolonged inflammatory state with compromised immune function and increased fatigue [75].

Much like AMPK, muscle-derived IL-6 works as a sensor of energy “status,” ultimately leading to glucose uptake, lowering of serum glucose levels, and lipid oxidation, all changes that improve global metabolic function and may synergize with cancer treatment with chemotherapy and RT. Muscle-based IL-6 also appears to directly activate AMPK in rat studies [76]. Finally, IL-6 stimulates the breakdown and oxidation of fat, further improving the global metabolic state [77].

Lastly, exercise stimulates the expression of brain-derived neurotrophic factor in muscle and brain, with the latter contributing to an increase in its serum concentration [78]. This mechanism has also been implicated in the beneficial effects of exercise on chronic fatigue [79].

## 5. Decrease in Radiation Treatment-Related Side Effects

While exercise provides an abundance of metabolic benefits potentially improving cancer-specific outcomes, it also appears to improve quality of life and side effects related to treatment with RT. Men with prostate cancer receiving three months of ADT were randomized to an intervention group that engaged in a resistance exercise program three times per week for a period of 12 weeks versus a control group [80]. Those men that engaged in resistance training experienced a significant reduction in fatigue and higher quality of life versus those in the control group. These men also experienced elevated levels of upper and lower body muscular fitness. These benefits were found to be independent of bodyweight and BMI, as they were similar between the groups after the study.

Other randomized data reveal similar findings in sedentary men on ADT for prostate cancer with exercise leading to decreased fatigue [81]. This study also revealed a durable response seen in exercise behaviors. Similar trials reveal that a supervised exercise training program yields additional benefits over material given to patients, with significant improvements in physical functioning, muscle strength, muscle mass [82], mental health, and sexual function [83].

The same group later randomized men receiving RT with and without ADT to usual care during RT versus aerobic exercise and resistance training [84]. Training regimens were carried out over a 24-week period and the primary endpoint assessment was fatigue, the most common side effect of RT. They found that resistance training improved aerobic fitness, quality of life, strength, and triglycerides when compared with usual care. Aerobic exercise improved both fitness and fatigue. Resistance training resulted in longer-term benefits, which may be consistent with the additional metabolic benefits derived from more intense activity.

Monga et al. randomized men to exercise group or a control group while undergoing RT for prostate cancer [85]. The men in the exercise group experienced improvements in cardiovascular fitness, flexibility, muscle strength, and overall quality of life and less fatigue.

## 6. Metabolic Management of Metabolic Dysregulation from ADT

While conclusive data support the usage of ADT in conjunction with RT for high-risk prostate cancer, toxicity from this treatment remains a concern for the treating physician. As discussed above, ADT works to reduce prostate cancer cell gene transcription through the reduction of circulating androgens capable of binding to the androgen receptor signaling proliferation [5]. Androgen deprivation is most commonly achieved with gonadotropin-releasing hormone (GnRH) agonists. Data have shown poorer results for men receiving ADT with a history of moderate-to-severe comorbidities [86]. Interestingly, ADT causes similar comorbidities and side effects from metabolic and physiologic alterations, including increased adipose tissue, cardiovascular disease, QT interval prolongation, insulin insensitivity, and diabetes [87]. As these changes could hinder both prostate-specific and overall health outcomes, methods to offset these side effects are of importance.

For instance, increased insulin resistance results in both elevated serum glucose and insulin, both of which can stimulate the IGF and other proliferative pathways, leading to increased cancer growth and resiliency from damage induced by RT [12]. Elevated insulin also correlates with increased risks of prostate cancer diagnosis [88] and recurrence after treatment [89]. Frequent exercise leads to persistently elevated levels of AMPK protein in skeletal muscle [52]. As the stimulation of AMPK increases insulin sensitivity [53] and decreases circulating glucose levels [54], these activities may serve to offset potential side effects of ADT while also enhancing the treatment effects of ADT.

The hormonal milieu induced by ADT may hinder the loss of fat mass seen during exercise, as corresponding results of exercise interventions reveal mixed results with regard to a reduction in body fat [90]. In a recent study by Nilsen et al. there were also no changes in fat mass between the control group and men performing high-intensity strength training over 16 weeks with 3 sessions per week performed in an undulating periodization style [91]. However, apart from changes in body composition, several beneficial effects of exercise have been reported on patients undergoing ADT [90].

For example, the Trans-Tasman Radiation Oncology Group randomized men receiving RT and ADT to six months of supervised exercise followed by an additional home-based exercise program or printed educational material [82]. Those men on the supervised exercise regimen experienced significant improvement in cardiorespiratory fitness performance, lower body physical function, self-reported physical functioning, appendicular skeletal muscle, and objective measures of muscle strength. Perhaps most importantly, these benefits persisted at one year in those on a home-based program.

In a similar study, 100 sedentary men with locally advanced or metastatic prostate cancer on long-term ADT were randomized to a three-month intervention of aerobic exercise and resistance training [81]. Functional Assessment of Cancer Therapy-Prostate (FACT-P) and Functional Assessment of Cancer Therapy-Fatigue (FACT-F) questionnaires at 3 and 6 months after the intervention revealed significant improvements in quality of life scores on FACT-P at 3 months and FACT-F at 3 and 6 months. Exercise levels were maintained in these men at the conclusion of the study.

Other data in men receiving ADT randomized to resistance training reveal significant reduction of fatigue, improved upper and lower body muscular fitness, and improved quality of life [80]. Again, the benefits in this study were seen even without improvement in body weight, body mass index, waist circumference, or subcutaneous skinfolds.

Men receiving ADT are interested in adding exercise regimens to their treatment. According to a survey, 79% of men are willing to participate in an aerobic exercise program during treatment and 81% are willing to engage in muscle-strengthening programs [92]. Men also preferred to exercise at home; flexible, spontaneous, and self-paced regimens were preferred. Due to the multiple physiologic, metabolic, and physical benefits of exercise, benefits that directly offset the potential toxicity of ADT, along with randomized evidence of benefit of both aerobic exercise and resistance training, exercise in conjunction with ADT and RT should be part of the standard of care in those men capable of safely engaging in these activities. Indeed many are now recommending that exercise interventions should be offered to all patients receiving ADT and should continue afterwards [93].

## 7. Moving Forward: Patient-Oriented Exercise

Many patients do not wish to exercise intensely or at a gym [94] and, unfortunately, only 19% of men receiving treatment for prostate cancer with ADT meet guidelines for weekly physical activity [92]. Although it seems that supervised activities provide more benefit than those that are unsupervised [95], tangible activities that are more likely to be adhered to by men with prostate cancer may also provide benefits. For example, according to data in the Harvard Health Publications, many activities provide a similar amount of calories burned which do not involve a gym or are not even considered as exercise by most people [96]. As listed in Table 1, many typical household activities burn a similar amount of calories as dedicated activities or exercise, and this can be emphasized to patients in an effort to increase overall activity levels.

Along these lines, discussions can ensue with patients to favor simple and tangible changes in activity levels to significantly increase overall daily activity levels. For instance, with a simple switch from three hours of television viewing per night (33 calories) to reducing television time to an hour and replacing those remaining hours with cooking, reading, and gardening in the morning (361 calories), patients can substantially increase their activity levels.

Additionally, advances in technology are allowing physicians to better track and quantify exercise habits for further discussions on methods to increase or improve activity levels. Current studies are underway at the University of Pittsburgh assessing activity levels during RT and mechanisms to increase these levels. As patient-centered technology and device designs increase, the opportunities to quantify patient activity and exercise levels are arming the physician with data that was unattainable even a few years ago. All aspects of activities, from intense exercise to low-intensity walking, appear to provide benefit [47]. High-intensity activity entails a strong myokine-mediated anti-inflammatory response and directly alters the metabolic environment via reductions in glucose, insulin, and the insulin pathway; if performed on a regular basis, this fosters an inhospitable setting for tumor growth and reduces the ability of cancer cells to overcome treatment-related damage [12]. Less intense activities improve antitumoral immune function [74] and help reduce adipose tissue via lipolysis, which can result in a global reduction of inflammation and secreted hormones that can fuel the growth of prostate cancer cells.

Such findings are encouraging for the treating physician and patient alike. Evidence that a variety of activities can improve a patient's overall and prostate-specific outcome provides options and flexibility to guide patients and increase the odds of success in following an exercise regimen. This may be a major challenge for patients in the midst of treatment when new physical and emotional difficulties serve as obstacles to adopting or continuing with exercise and healthy habits.

## 8. Conclusions

Based on the data presented above, a prudent exercise and activity goal for the prostate cancer patient to increase his chance of cure would be a multifaceted approach to reduce overall and central adipose tissue deposition and to mitigate circulating levels of inflammation, insulin, and detrimental sex hormones. Further studies to assess the most efficacious techniques are needed. Activity levels, ranging from walking to more intense activities and exercise regimens, provide unique benefits. Randomized data continue to accumulate regarding the positive effect that exercise has on treatment outcomes for men with prostate cancer and the implementation of exercise during and after treatment for prostate cancer should be part of the standard of care.

The radiation oncologist is provided with a unique opportunity to reiterate healthy lifestyle approaches and modifications due to the extensive time spent with patients on a weekly basis during treatment. Prostate cancer is one of the more prolonged treatment regimens, and the oncologist is given multiple opportunities to suggest and help implement these exercise and lifestyle changes. Our challenge as clinicians is to create opportunities to guide, encourage, and support patients as they adopt healthy lifestyle behaviors for physical activity. As demonstrated by this review, these approaches are increasingly evidence based and mechanistically understood.

## Figures and Tables

**Table 1:** 

	Calories expended^*∗*^
*Gym activities*	
Weight lifting (general)	133
Water aerobics	178
Stretching, Hatha yoga	178
Calisthenics (moderate)	200
Riders (i.e., HealthRider)	222
Aerobics (low impact)	244
Stair-stepper machine (general)	266
Teaching aerobics	266
Weight lifting (vigorous)	266
Aerobics, step (low impact)	311
Aerobics (high impact)	311
Bicycling, stationery (moderate)	311
Rowing, stationery (moderate)	311
Calisthenics (vigorous)	355
Circuit training (general)	355
Rowing, stationery (vigorous)	377
Elliptical trainer (general)	400
Ski machine (general)	422
Aerobics, step (high impact)	444
Bicycling, stationery (vigorous)	466

*Outdoor activities*	
Planting seedlings and shrubs	178
Raking lawn	178
Sacking grass or leaves	178
Gardening (general)	200
Mowing lawn (push, power)	200
Operate snow blower (walking)	200
Plant trees	200
Gardening (weeding)	205
Carrying and stacking wood	222
Digging and spading dirt	222
Laying sod/crushed rock	222
Mowing lawn (push, hand)	244
Chopping and splitting wood	266
Shoveling snow (by hand)	266

*Home and daily life activities*	
Sleeping	28
Watching TV	33
Reading (sitting)	50
Standing in line	56
Cooking	111
Child care (bathing, feeding, etc.)	155
Food shopping (with cart)	155
Moving (unpacking)	155
Playing w/kids (moderate effort)	178
Heavy cleaning (wash car and windows)	200
Child games (hopscotch, jacks, etc.)	222
Playing w/kids (vigorous effort)	222
Moving (household furniture)	266
Moving (carrying boxes)	311

*Home repair*	
Autorepair	133
Wiring and plumbing	133
Carpentry (refinish furniture)	200
Lay or remove carpet/tile	200
Paint, paper, remodel (inside)	200
Cleaning rain gutters	222
Hanging storm windows	222
Paint house (outside)	222
Carpentry (outside)	266
Roofing	266

^*∗*^In 30 minutes for a 185 lb man. Table created with data from Harvard Health Publications.
